# “Descriptive Risk-Averse Bayesian Decision-Making,” a Model for Complex Biological Motion Perception in the Human Dorsal Pathway

**DOI:** 10.3390/biomimetics7040193

**Published:** 2022-11-07

**Authors:** Khashayar Misaghian, Jesus Eduardo Lugo, Jocelyn Faubert

**Affiliations:** 1 Sage-Sentinel Smart Solutions, 1919-1 Tancha, Onna-son, Kunigami-gun, Okinawa 904-0495, Japan; 2 Faubert Lab, School of Optometry, Université de Montréal, C.P. 6128, Montreal, QC H3C 3J7, Canada; 3 Facultad de Ciencias Físico-Matemáticas, Benemérita Universidad Autónoma de Puebla, Av. San Claudio y Av. 18 Sur, Col. San Manuel Ciudad Universitaria, Puebla Pue 72570, Mexico

**Keywords:** biological motion, Bayesian, dorsal pathway, hierarchical simulation model

## Abstract

Biological motion perception is integral not only to survival but also to the social life of human beings. Identifying the underlying mechanisms and their associated neurobiological substrates has been a matter of investigation and debate for some time. Although, in general, it is believed that the integration of local motion and dynamic form cues in the brain empowers the visual system to perceive/recognize biological motion stimuli, some recent studies have indicated the importance of dynamic form cues in such a process. Inspired by the previous neurophysiologically plausible biological motion perception models, a new descriptive risk-averse Bayesian simulation model, capable of discerning a ball’s direction from a set of complex biological motion soccer kick stimuli, is proposed. The model represents only the dorsal pathway as a motion information processing section of the visual system according to the two-stream theory. The stimuli used have been obtained from a previous psychophysical study on athletes in our lab. Furthermore, the acquired psychophysical data from that study have been used to re-enact human behavior using our simulation model. By adjusting the model parameters, the psychometric function of athlete subjects has been mimicked. A correlation analysis between human and simulation data shows a significant and robust correlation between angular thresholds and slopes of the psychometric functions of both groups. Although it is established that the visual system optimally integrates all available information in the decision-making process, the results conform to the speculations favoring motion cue importance over dynamic form by testing the limits in which biological motion perception only depends on motion information processing.

## 1. Introduction

Humans’ robust ability to recover information (e.g., identity or type of activity) about a moving living thing from sparse input is known as biological motion perception. Such a sparse input was created and introduced by Johansson in 1973, using only light points placed on an individual’s strategic joints. Biological motion perception is critical to the survival and social interactions of humans and primates and plays a significant role in their activities. In this regard, there has been an emphasis on the visual analysis of human action in multiple studies with a primary focus on the kinematic information of the movements (such as the type of activity and emotional states), the motor role in the perception of actions, and the neural mechanisms (Blake and Shiffrar, 2007; Giese and Poggio, 2003) [1,2].

The many psychophysical, neurophysiological and functional imaging experiments conducted on movement perception has resulted in a wide range of experimental data and findings. Activation of the “dorsal pathway,” specialized in motion information processing, the forms pathway (ventral pathway) and where the two streams converge at the superior temporal sulcus (STS) are counted as the highlights of these discoveries, as mentioned previously (Beintema and Lappe, 2002; Mather et al., 1992) [3,4].

Furthermore, significant accumulation of experimental data and the necessity for a compelling theoretical framework has emphasized the specific demand for a neurophysiologically plausible computational model for biological motion perception (Blake and Shiffrar, 2007) [1].

Based on the assumption that the visual system stores prototypical patterns in the perception/cognition process, a hierarchical feed-forward model has been proposed (Giese and Poggio, 2003) [2]. The model entailed two parallel processing modules simulating the ventral (form) and dorsal streams (optic flow). The study concluded that local motion analysis taking place in the dorsal pathway is the most critical factor in pattern detection (Blake and Shiffrar, 2007; Casile and Giese, 2005; Giese and Poggio, 2003) [1,2,5], contradicting an earlier study by Beintema and Lappe (Beintema and Lappe, 2002) [3]. Only recently has it been suggested that a multitude of contingencies are at work simultaneously to integrate either local motion or dynamic form analysis to make the perception/recognition of actions in the presence of internal and external noise as robust as possible (Blake and Shiffrar, 2007) [1].

While behavioral evidence implies the existence of low-level filters capable of capturing motion cues to detect biological motion as pure-motion detection mechanisms (Chang and Troje, 2008; Troje and Westhoff, 2006) [6,7], developmental studies also show a natural predilection towards biological motion in human infants and newborn chicks while suggesting the lack of such sensitivity to biological form cues. To be precise, unlike motion, the visual system processes the biological and non-biological dynamic form in the same fashion, and there is no specialized substrate in the brain for dealing explicitly with biological form (Bardi et al., 2011; Vallortigara et al., 2005) [8,9]. Thereafter, in 2014, to validate the behavioral results from their study and hypothesis, Thurman and Lu proposed a Bayesian template-matching model, which integrated form features of the stimuli using some weighting scheme, asserting that the dynamic form analysis pathway (ventral pathway) works for both biological and non-biological motion in a similar Bayesian fashion. This result suggests the absence of a specialized substrate for the processing of dynamic biological form (Thurman and Lu, 2014) [10].

Further investigations of the neural correlates involved in the perception of body movement show an extensive cortical network (Grosbras et al., 2012) [11]. Even though the biological motion perception incorporates both form and motion (Kourtzi et al., 2008) [12] and, therefore, the cortical regions linked to both cues get activated, it is unclear that the contribution of those areas is all of causal nature (Gilaie-Dotan et al., 2015) [13]. Recently, to address whether the activations of the ventral pathway during biological motion detection are functionally integral to the perception process, one study examined six patients with focally (compromised) injured ventral visual cortex in multiple regions. Not only did they all manage to recognize the point light stimuli, but their thresholds were also not significantly different from the control group’s thresholds. More interestingly, they significantly outperformed subjects with impairment in other regions critical to biological motion perception (Gilaie-Dotan et al., 2015) [13].

In regard to the question of modeling and simulating this phenomenon more explicitly, one could always picture the detection of biological motion as a sequential decision-making task. As with many other natural scenarios, biological motion perception also occurs in the presence of uncertainty, which stems from the inherent uncertainty of the subject’s generative model and the noise of the input process (Bitzer, Park, Blankenburg, and Kiebel, 2014) [14]. Uncertainty begets risk, so it is essential to deem human decision making to be subjected to this factor and, therefore, not always economically rational. In that light, a more plausible model could most certainly benefit from taking the risk factor into account (Kahneman and Tversky, 2013) [15]. In recent years, the risk-sensitive decision-making problem has been brought to researchers’ attention and been investigated in different areas including neuroeconomics and cognitive sciences (Braun et al., 2011; Dayan and Niv, 2008; Nagengast et al., 2010; Niv et al., 2012; Shen et al., 2014) [16,17,18,19,20].

Here, we intend to propose a feed-forward risk-sensitive Bayesian simulation model. The suggested model is hierarchical and appropriates the earlier assumption of stored prototypical patterns in STS located in the temporal lobe of the brain. Moreover, to model the motion pattern neurons, which are the decision-making neurons and also believed to be located in STS, a dynamic model called the mutual inhibition network was utilized (Lugo et al., 2018) [21]. The presented model has been challenged with a stimulus of higher complexity, namely a soccer kick, only to detect the direction of the ball from the subject’s point of view. Furthermore, as for the proof of concept of the Gilaie and Dotan study in 2015, the ventral pathway was implemented (Gilaie–Dotan et al., 2015) [13]. Finally, the behavioral data that had been collected previously in our lab was used to validate the performance of the proposed model, in so far as the model has been tuned to different modes only to replicate the behavior of 11 athlete subjects. The simulated psychometric function parameters show a significant correlation with those of athlete human subjects (Romeas and Faubert, 2015) [22].

## 2. Model

**Figure 1:**
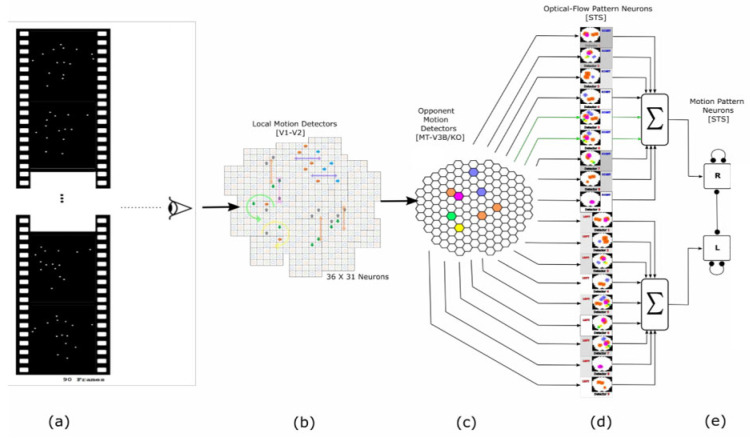
Schematic of the model in one hypothetical point in time, from left to right: (**a**) the reel of biological motion stimulus, (**b**) local motion detectors as an ensemble of 1116 neurons positioned in a 36 by 31 arrangement, that fire due to motions they experience during two consecutive frames, represented by the cells with color-filled arrows (blue: right, orange: left, grey: up, and green: down). The larger, two-headed, colorful arrows are drawn to display the types of opponent motions that would be sensed at the next level (cyan: horizontal expansion, orange: vertical expansion, and magenta: vertical contraction). (**c**) Opponent motion detectors as an ensemble of 100 neurons to detect horizontal expansion, horizontal contraction, vertical expansion, and vertical contraction. The activated detectors are marked with color-filled hexagons with their corresponding color (cyan: horizontal expansion, orange: vertical expansion, and magenta: vertical contraction). (**d**) Optical-flow pattern detectors as an arrangement of 18 neurons following one-dimensional mean-field dynamics, each neuron incorporates a statistical template (displayed as a colorful map) that represents a specific part of the manifold of the kicking sequences (for example neuron number two contains a template for the seconds 11 to 20 of the kick-to-right sequence, while neuron number 10 would have a larger instantaneous input for the seconds 1 to 10 of the kick to the left stimulus). Green arrows highlight the contribution of two cells to the evidence integration at that hypothetical point due to the similarity of the evidence signal and their template. (**e**) Thresholding stage, comprising two decision neurons for the right and left decisions (marked by capital letters R and L on the square cells with soft edges) follow our mutual inhibition dynamics receiving their corresponding inputs from the integration stage. The straight and curve lines with rounded heads highlight the inhibitory interaction between the neurons and the auto-inhibition, respectively. No activity could be seen by either of the neurons since at that hypothetical point in time, neither had made a decision yet.

The neural model devised for our simulations is inspired by the biologically plausible model proposed by Poggio and Giese (2003) and by Cassile and Giese (2005) (Casile and Giese, 2005; Giese and Poggio, 2003) [3,5]. Our simulation model appropriates three assumptions of the models, as mentioned earlier: 1. Dorsal stream (Optic flow) which, as with other visual pathways, consists of hierarchies of neural detectors to extract optic-flow features. 2. This model adopts a predominantly feed-forward architecture. 3. The visual system stores prototypical patterns and uses them for perception/recognition (Figure 1)

The neural hierarchy of the dorsal stream is as follow:

### 2.1. Local Motion Energy Detectors

These detectors are sensitive to different motion directions and have small receptive fields (≈0.4 deg). For the present study, the simulations have implemented receptive fields which are sensitive to four different directions: right, left, up and down and, for the sake of simplicity, no diagonal direction has been implemented. These detectors have been deployed in a 36 × 31 assembly of receptive fields according to Smith et al. (1994). It has been reported that these selectively acting neurons reside in the monkey visual cortex in area V1/2 and area MT (A. T. Smith and Snowden, 1994) [23].

To simplify things, we calculated the optical flow of every two consecutive frames of the stimulus so that the activity of all the assigned motion detectors in the 36 × 31 assembly could be approximated at each time point (every 500 ms) using the obtained vector field. A more detailed explanation of the implementation of this level can be found in the study of Casile and Giese [5].

### 2.2. Opponent-Motion Detectors

These detectors are sensitive to opponent motions such as expansions, contractions, and rotations. For example, a neuron specialized in vertical contraction detection gets activated by the occurrence of such an opposite motion in the two adjacent subfields located in its receptive field (A. T. Smith and Snowden, 1994) [23]. The opponent motion detector pools the responses of the local motion detectors of the same direction preference into one subfield using a maximum operator. In the case of vertical contraction, the detector pools the rightward motion in the left subfield and leftward motion from the adjacent subfield. The output of the opponent motion is made up of the multiplication of these maxima (The square root of this multiplicative pooling) (Allman et al., 1985) [24]. Utilization of the/a maximum operator in the opponent motion-sensitive neurons simulation is rooted in the discovery of the same sort of computation in the visual cortex of monkeys and cats (Gawne and Martin, 2002; Lampl, Ferster, Poggio, and Riesenhuber, 2004) [25,26]. Moreover, the pooling process entails a spatial invariance within the respective receptive fields (Riesenhuber and Poggio, 1999) [27].

Imaging studies suggest that opponent-motion detectors probably exist in the kinetic occipital area (KO/V3B) of humans (Orban et al., 1995; Orban et al., 1992) [28,29].

Similar to Cassile’s 2005 study, we implemented the four types of opponent motion—vertical and horizontal, contraction and expansions—using 5 × 5 assemblies of detectors to generate 100 simulated features at each time point (every 500 ms). For more descriptive details one must refer to (Casile and Giese, 2005) [5].

### 2.3. Complex Global Optical-Flow Patterns

The third hierarchy level is made up of neurons capable of discerning momentarily complex optic flow patterns. The other critical factor to which these neurons must be responsive is the temporal order of the input that they are receiving. In other words, each detector is tuned to a certain optic flow pattern of a certain temporal order. In previous attempts to model optic flow detectors with this characteristic, a network of laterally coupled neurons has been proposed. It is through these asymmetrical connections that the active neuron at one moment excites the neurons tuned to the future optic flow patterns and inhibits the rest of the detectors encrypting earlier patterns (Mineiro and Zipser, 1998) [30]. In this manner, the assumed dynamic of the optic flow pattern neuron sensitive to the ith frame (the optic flow that comes from the i−1 and ith frames) of one stimulus sequence is as follow (Casile and Giese, 2005) [5]:

(1) $$\tau_{OFP}{\dot{H}}_{i}\left(t\right)=-H_{i}\left(t\right)+G_{i}\left(t\right)+\underset{m}{\sum }w\left(i-m\right)f\left(H_{i}\left(t\right)\right)$$

where $H_{i}\left(t\right)$ is the activity of the ith neuron, the $\tau_{OFP}=150\;\mathrm{ms}$ is the time constant of the global optic flow pattern detection dynamic, w(m) is an asymmetrical weight kernel, f(H) is a step threshold function, and $G_{i}\left(t\right)$ is considered the instantaneous feed-forward input of the neuron. As mentioned before, one of the fundamental assumptions about the model is the prototypical matching performed by the neuron. It is only the result of this template matching process that constitutes the aforementioned feed-forward input. In previously proposed models, for each key feature vector derived from the stimulus video sequence, namely template, a Gaussian radial basis function has been designated and tuned to it. Thus, when the detector receives its input from the previous level, depending on how similar it is to the center of the corresponding Gaussian function, the instantaneous feed-forward input gets generated. For a detailed description of this, the reader is referred to Giese and Poggio (2003) and Casile and Giese (2005) (Casile and Giese, 2005; Giese and Poggio, 2003) [2,5]. While our model holds the exact laterally connected dynamics for the optical flow detectors, it uses far fewer neurons and a different strategy to generate the instantaneous feed-forward input, $G_{i}\left(t\right)$.

In our model, the feed-forward input $G_{i}\left(t\right)$ is deemed to be a product of a multiclass Bayesian classification scheme. Here, the most classical minimization of classification errors did not seem to serve the purpose; instead, minimizing the average risk method, which includes different significances for different errors, shows more efficiency. To be more precise, false classification of the represented frame into one of the future vital feature-vectors must have less gravity compared to one related to an older template (Theodoridis, 2010) [31]. The logic behind it can be explained by the goalkeeper example; meaning that if a goalkeeper decides that the frame observed in a scene belongs to one of the future states of the sequence, the chance to save the ball is less compromised as opposed to classifying that scene into one of the earlier-encoding templates.

The same stimuli have been used in previous experiments in our lab (Romeas and Faubert, 2015) [22]. Each stimulus sequence comprises 90 frames. For every two consecutive frames (after passing through the first two levels), a feature-vector of 140 elements would be generated and fed to the optical flow pattern detection stage. There exists a stimulus for every angle of deviation from the center in order to train or test the model. We considered nine stages for each kicking sequence. Our classification problem consists of 18 classes, nine classes for the rightward kick and nine for the leftward. Each class represents one specific stage of the kicking process, i.e., the first class associated with right-ward kick means we are in the first stage of the kicking process (first ten frames) and the third class associated with a left-ward kick is the right decision when the stimulus reaches somewhere between frames 31 to 40. Therefore, our problem is an 18-class, $\omega_{i},\;i=1,\ldots ,18$ classification problem, where $R_{j},\;j=1,\ldots ,18$ makes up the regions of the feature space. An error happens when the feature-vector u which pertains to the region $R_{i}$ gets misclassified in class $\omega_{k}$ while i≠k and so a loss term, $\lambda_{ki}$, will be assigned to this incorrect decision. In this manner, a loss matrix could be formed whose element $\lambda_{ki}$ constitutes the penalty for action k (here: classification in class $\omega_{k}$) when the actual state is i (the feature-vector fed to the layer). It can be shown that the average risk is minimized when (Theodoridis, 2010) [31]:

(2) $$u\in \;R_{i}\;if\;\overset{18}{\underset{k=1}{\sum }}\lambda_{ki}p\left(u|\omega_{k}\right)p\left(\omega_{k}\right)<\overset{18}{\underset{k=1}{\sum }}\lambda_{kj}p\left(u|\omega_{k}\right)p\left(\omega_{k}\right)\;\lambda_{ll}=0,\;\forall \;j\neq i$$

which indicates, that u originates from the region $R_{i}$ when it has the lowest weighted sum and classifies in class $\omega_{i}$.

$p\left(u|\omega_{k}\right)$ is the likelihood of the feature-vector given that the class $\omega_{k}$, and $p\left(\omega_{k}\right)$ is the prior probability of the class $\omega_{k}$. In our model, we assume that the likelihood of feature-vectors of each region $R_{i}$ follows a Gaussian distribution $N\left(\mu_{i},\Sigma_{i}\right)$, in which, $\mu_{i}$ is the mean vector, and $\Sigma_{i}$ is the covariance matrix. Moreover, the priors, $p\left(\omega_{k}\right)$, are predefined for each class $\omega_{k}$ separately where $\overset{18}{\underset{k=1}{\sum }}p\left(\omega_{k}\right)=1$.

In this manner, the detector tuned to class $\omega_{i}$ receives a positive non-zero feed-forward input, $G_{i}\left(t\right)$, at each time step whenever u(t) belongs to the feature region, $R_{i}$.

To describe it at the cellular level, when one input matches the saved template of one neuron, all other neurons with different classes see that as a sizeable, weighted quantity added to their risk sum while the loss term $\lambda_{ii}=0$ relieves the matching neuron from adding that large signal to its risk sum. In other words, the neuron with the matching template inhibits the feed-forward input of other neurons.

The consensus is that the complex optic flow pattern neurons are likely to be found in disparate areas of the superior temporal sulcus (Decety and Grèzes, 1999; Oram and Perrett, 1994; Perrett et al., 1985; Vaina et al., 2001) [32,33,34,35].

### 2.4. Complete Biological Motion Pattern Detectors (Motion Pattern Detectors)

Discrimination of complete biological motion patterns occurs in motion pattern neurons, which make up the fourth and highest level of the model. The complete biological actions in our study are composed of leftward and rightward kicks. The sum of the activities of the optic flow pattern detectors that belong to one particular action serves as an input to the motion pattern detector associated with that very action. It is the activities of these motion pattern detectors which constitute the decision response or more generally the behavior of the biological motion detection system (Casile and Giese, 2005; Giese and Poggio, 2003) [2,5]. Moreover, imaging studies have accounted for the possibility of the existence of motion pattern neurons in STS (Grossman et al., 2000; Vaina et al., 2001) [35,36], and perhaps also in FFA (Grossman et al., 2000) [36].

A non-linear, excitatory and inhibitory network has been adapted to simulate these motion pattern detectors, which had initially been used to describe neuronal polarity under various circumstances (Lugo et al., 2018) [21]. In this mechanism, which is known as mutual or global inhibition, the element with the highest excitatory input suppresses the activity of those whose activities have not passed their thresholds in a nonlinear and reciprocated fashion (Lugo et al., 2018; Wilson, 1999) [21,37].

The fact that mathematical models similar to the mutual inhibition model have shown success in the simulation of humans’ decision neuronal networks (Wilson, 1999) [37] is the reason behind this choice of model.

### 2.5. Robust Mutual Inhibition Model

Initially, the mutual inhibition model (Lugo et al., 2018) [21] explains the response of decision making neurons using the following nonlinear dynamic below:

(3) $$\;\tau \frac{dT}{dt}=-T+S\left(P_{T}\left(D\right)\right)$$

(4) $$\tau \frac{dD}{dt}=-D+S\left(P_{D}\left(D,T\right)\right)$$

where T is the activity of the first neuron to get excited by the activity of the previous hierarchy level and D is the activity of the remaining neurons. τ is a time constant and S() is a modified Michaelis–Menten function (Wilson, 1999) [37] which is especially useful in designing excitatory–inhibitory networks (Lugo et al., 2018) [21]:

(5) $$S\left(P\right)=\left\{\begin{array}{c} \frac{MP^{2}}{\sigma^{2}+P^{2}}\;P\geq 0\\ 0\;P<0 \end{array}\right\}$$

where M is the maximum information threshold for the excitatory–inhibitory activity and σ almost always marks the information threshold point where the function hits half of its maximum. $P_{T}$ and $P_{D}$ are the information thresholds available to T−type and D−type neurons, respectively:

(6) $$P_{T}\left(D\right)=E_{T}-kND$$

(7) $$P_{D}\left(D,T\right)=E_{D}-k\left(N-1\right)D-kT$$

where N is the number of neurons and the constant k is the inhibitory feedback gain. Also, $E_{T}$ and $E_{D}$ represent the external inputs generated from the previous hierarchy level. The number of equations to solve depends on how many decision-making neurons are involved in the process. For instance, if we wanted decision-making agents to pick one choice out of N choices, we would need to solve one Equation (6) and N−1 Equation (7). Thus, N=2, since, in our model, the decision is between left and right kicks. For more information and mathematical details, one must refer to Lugo et al. (2018) (Lugo et al., 2018) [21].

The original mutual inhibition model dictates that only the non-negative information thresholds, $P_{T}$ or $P_{D}$, would contribute to the activity of the decision neurons and, when negative, the neuron activity attenuates exponentially according to the linear first-order dynamic that it follows in the absence of any input and interconnection between other neurons. Although this implementation maintains some degree of robustness, it falls short when facing the high variation signals coming from the third hierarchy layer. To reduce the level of sensitivity, we modified the system to neglect the negative changes. In other words, when neurons are disconnected as a result of negative information thresholds, detectors’ activities will be as follows:

(8) $$\;\tau \frac{dT}{dt}=\left\{\begin{array}{c} -T\;T\leq 0\\ \;0\;T>0 \end{array}\right\}\;$$

(9) $$\tau \frac{dD}{dt}=\left\{\begin{array}{c} -D\;D\leq 0\\ \;0\;D>0 \end{array}\right\}$$

### 2.6. Modeling of the Internal Noise

To simulate uncertainty in the decision-making process, we assumed that the output of each optic flow pattern neuron is drawn from a Gaussian distribution, $N\left(H_{i}\left(t\right),∆t\delta^{2}\right)$, where $H_{i}\left(t\right)$ is the ideal activity of the optic flow neuron in the absence of the added internal noise of the variance, $\delta^{2}$. In our model, this implementation can be construed as the generative input process of the fourth layer due to the physiological noise in the visual pathway. It also can be shown that, in the particular case of the constant priors for generating the feed-forward input $G_{i}\left(t\right)$, such exercise mirrors the uncertainty in the internal generative models of the third layer. In this case, the added noise represents the error between the internal generative model and the feature input that the decision-making agent receives.

## 3. Methods

All implementations of the simulation model were executed in Matlab, and for the data fitting and statistical analyses, R Studio platform was used.

### 3.1. Stimuli and Data

To simulate the same conditions of the psychophysics study (Romeas and Faubert, 2015) [22] for which we propose a simulation model, we adopted the same original point light soccer kick captured by Mixamo studio. The stimulus comprises 15 dots representing the head and the human body’s major joints (shoulders, hips, elbows, wrists, knees, and ankles). The stimulus is composed of 90 frames with a duration length of 4.5 s. By rotating the original stimulus around the *Z*-axis, we were able to create the stimuli for leftward and rightward point-light soccer kicks with different angles. In the psychophysics study, subjects were exposed to the stimuli with deviations of 2°, 4°, 8° and 15° angles either towards the left-hand side or right-hand side of the viewer. For training and cross-validation of the model, the utilized data are comprise all shooting angles within the range of 1° to 20°. This range is the angular range in a penalty kick from the goalkeeper’s point of view. As a real-world example, a penalty kick resembles a wide range of situations. Nonetheless, in a regulated and constrained condition, it provides a framework to estimate an angular range within which one can assume the human brain has been trained. In other words, we believe that this angular range is the approximate range that constructs the prototypical patterns in one’s visual system.

A k-fold cross-validation (k = 5) procedure was used to validate our model (Jung and Hu, 2015) [38]. Thenceforth, the model was trained in the range of 7° to 20° and, moreover, tested for angles 2°, 4°, 8° and 15° to recreate the behavioral test conditions.

### 3.2. Local Motion Energy and Opponent Motion Neurons

The methods to implement the 1st and the 2nd hierarchy level of the present simulation model have been borrowed from the previous studies (Casile and Giese, 2005; Giese and Poggio, 2003) [2,5].

### 3.3. Optic Flow Pattern Neurons

For each direction (left or right), we installed nine optical flow pattern detectors. Each detector is selective for 10 consecutive frames out of 90; for example, neuron $H_{1}^{Left}$ is selective for frames 1 to 10 of the left side shooting and $H_{6}^{Right}$ is selective for the frames 41 to 50 of the right-side shooting. Each neuron incorporates an internal generative model, $p\left(u|\omega_{k}\right),\;k=1,\ldots ,18,$ assumed to be of the Gaussian form, $N\left(\mu_{k},\Sigma_{k}\right)$. The mean, $\mu_{k}$, and covariance matrix, $\Sigma_{k}$ of each template are computed using feature vectors derived from 10 frames of multiple stimuli with different degrees of deviation. For instance, $H_{6}^{Right}$ is trained using feature vectors from frames 61 to 70 of the shots with 7° to 20° of deviation to the right-hand side of the observer. Concisely,$\;H_{6}^{Right}$ is supposedly selective for frames 61 to 70 regardless of the deviation of the shots.

While the feed-forward input to each optic flow neuron is derived from the previous layer output, the dynamic of the neurons of this level, following (1), is solved using Euler’s method. To provide the input for the next hierarchy level, an independent Gaussian noise was added to the activity of each of the optical flow detectors.

**Figure 2:**
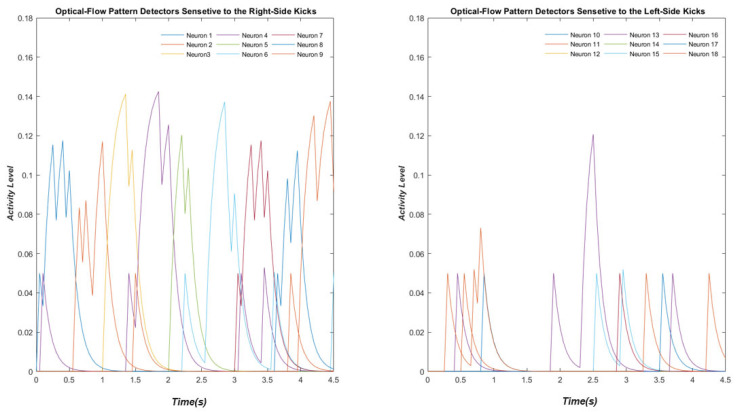
The activity of these neurons in the absence of the internal noise to the stimulus representing a kick with 9° of deviation to the right. Neurons 1 to 9 are responsive to the right-side kicks and neurons 11 to 18 are sensitive to the left-side kicks.

Below, the activity of these neurons in the absence of the internal noise to the stimulus representing a kick with 9° degrees of deviation to the right is demonstrated (Figure 2).

### 3.4. Motion-Pattern Neurons

At the decision-making layer, two motion pattern neurons were implemented, one for the leftward kick motion and the other for the rightward kick motion. As described in the previous section we modeled the dynamic of these detectors using a robust mutual inhibition method. The fourth-order Runge–Kutta method was utilized to solve the nonlinear system dynamics.

**Figure 3:**
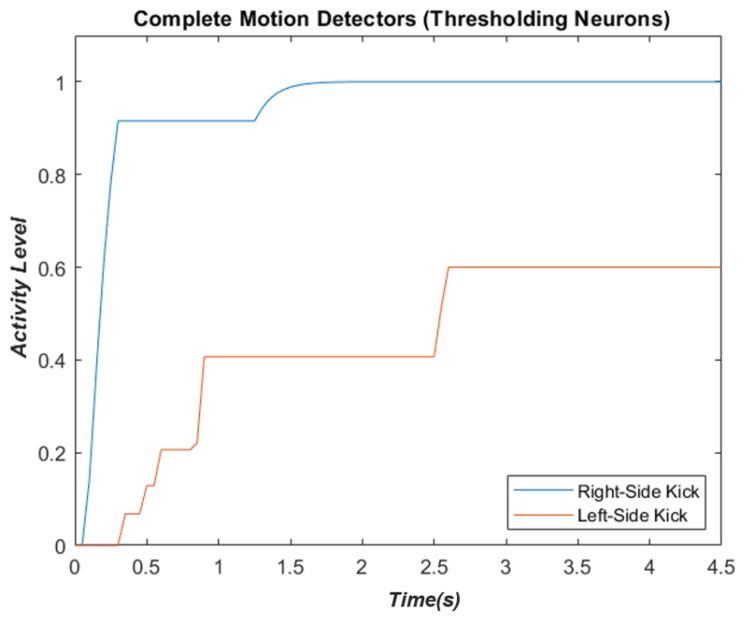
The neuron responsive to the right-side kick (blue) is highly activated, while the inhibition in the other neuron is evident.

Furthermore, the activity of these decision-making neurons as members of the thresholding stage when the stimulus is a sequence of a 9° degrees to the right kick, while the internal noise is not present, is depicted in Figure 3, below:

### 3.5. Simulating Human Behavior

In their behavioral study, Romeas and Faubert (2015) utilized a forced-choice paradigm task to decide the direction of the ball (left or right) by relying solely on the biological motion signal (Romeas and Faubert, 2015) [22]. Each subject was exposed to a total of 1080 randomized stimulus sequences of left and right shots with deviations of 2°, 4°, 8° and 15° angles (120 times for each angle at each side). Accordingly, for each subject, a psychometric function to relate human behavior to the angular deviation was determined (Romeas and Faubert, 2015) [22]. Here, to simulate those performances, three parameters of our simulation model were adjusted to mimic the behaviors of 35 athlete subjects from the psychophysical study.

Just as with the behavioral study, for each angle and side, the simulation model was exposed to the particular stimulus 120 times in order to generate an error percentage quantity. Additionally, this was repeated 30 times, and the corresponding psychometric function was determined using the simulated results.

The three variables for tuning the model to 35 athlete subjects were:

1. The standard deviation of the added internal noise, δ.
2. The time constant, τ.
3. The inhibitory feedback gain, k.

## 4. Results

While the previously proposed models, trained with similar data, performed very poorly for detecting the point-light kicking sequences, our model efficiently showed outstanding performance facing the stimuli. The five-fold cross-validation of our model resulted in an average success of 87.5%.

**Figure 4:**
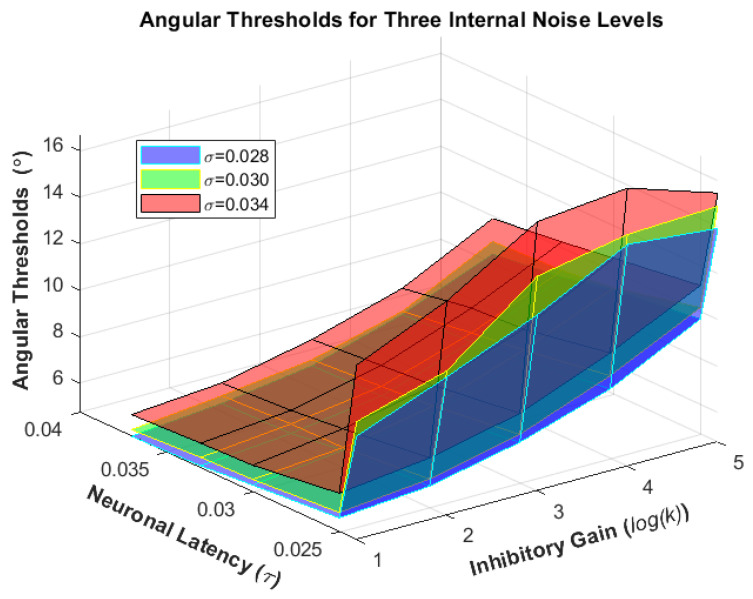
The resulting psychometric function angular thresholds when running the model for exemplary ranges of neuronal latency (τ=0.024, 0.025, 0.03, 0.033, 0.037 s) and inhibitory gain (k=2, 4, 8, 16, 32) for three noise levels (δ=0.028, 0.030, 0.034).

**Figure 5:**
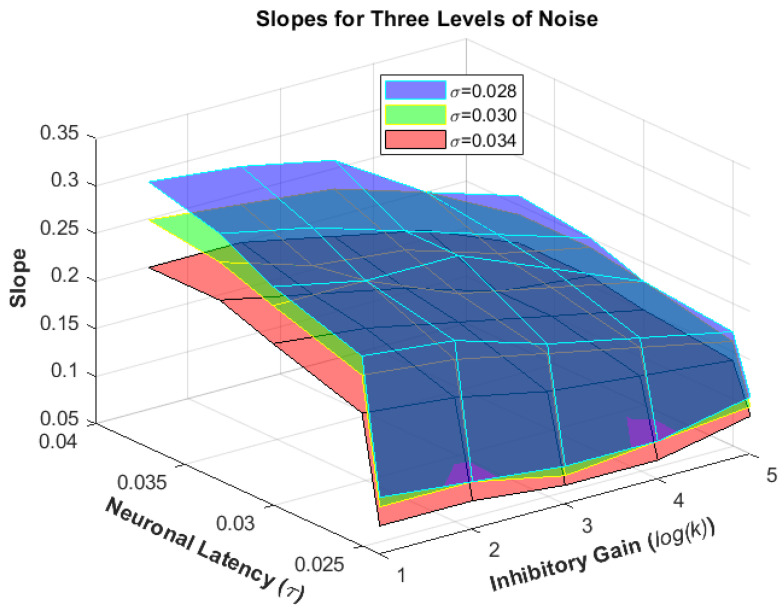
The resulting psychometric function slopes when running the model for exemplary ranges of neuronal latency (τ=0.024, 0.025, 0.03, 0.033, 0.037 s) and inhibitory gain (k=2, 4, 8, 16, 32) for three noise levels (δ=0.028, 0.030, 0.034).

The model performance demonstrated remarkable robustness in the presence of a wide range of imposed internal noise, δ. Besides noise being a prominent adjuster of our model, the degree of inhibition occurring between two decision making neurons, represented by k, along with their intrinsic latency, represented by τ, prove to be critical factors to bring the model into different functional states. Grid search computation was performed for different ranges of internal noise, δ, mutual inhibition gain, k, and intrinsic latency of the decision neurons (thresholding stage), to generate the results. Here we present a part of the results from solving the model for different parameters as an effort to get an insight into how each parameter could contribute to the model’s decision-making behavior (Table 1). Increase in neurons’ dynamic time constant, τ always results in better performance, meaning lower angular thresholds and steeper slopes, while an increase in the inhibitory gain, k shows a different trait (Figure 4 and Figure 5).

At first, the increase of inhibition gain k, leads to a deterioration in performance (higher threshold, flatter slope) but this change after k passes the value of eight. However, the phenomenon seems less evident for δ = 0.030. At that noise level, the angular thresholds do not decrease but rather increase at a lower rate; however, one must notice the slopes taking on a new trend wherein they become steeper, which can mean only that a higher accuracy is being reached in smaller angular deviations.

Unsurprisingly, the betterment of the performance does not come free of cost. Analyzing the activity of decision neurons shows it takes far more time for the winning neuron to reach the highest point of its activity when the inhibitory gain, k, is too large, and this can only be interpreted as longer processing time.

### Human Results vs. Simulation Results

By adjusting the parameters mentioned above: internal noise δ, mutual inhibition time constant τ, and inhibitory gain k, the behaviour of 35 athlete subjects was mimicked. The parameters and the associated simulated angular thresholds and slopes versus the experimental values are reported below in Table 1. Subjects were grouped by similar angular thresholds and slopes and each group was simulated by one set of parameters. Such an approach helps to acquire a more generalized understanding of subjects’ behaviors as groups.

**Figure 6:**
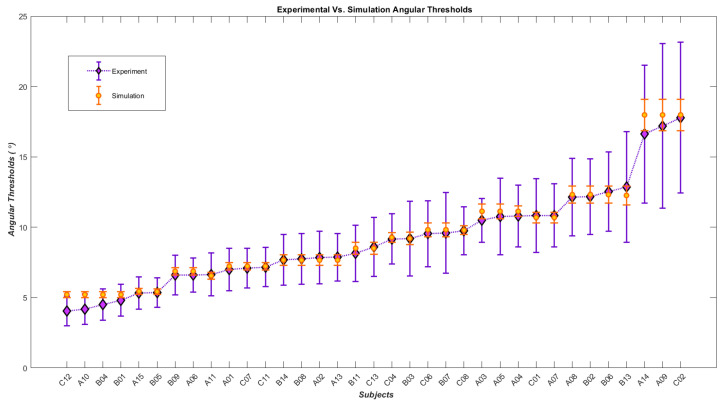
Diamonds represent the angular thresholds (75%) calculated from the psychometric function of the subjects in the experimental tests and the black dots display the angular thresholds generated by simulation.

**Figure 7:**
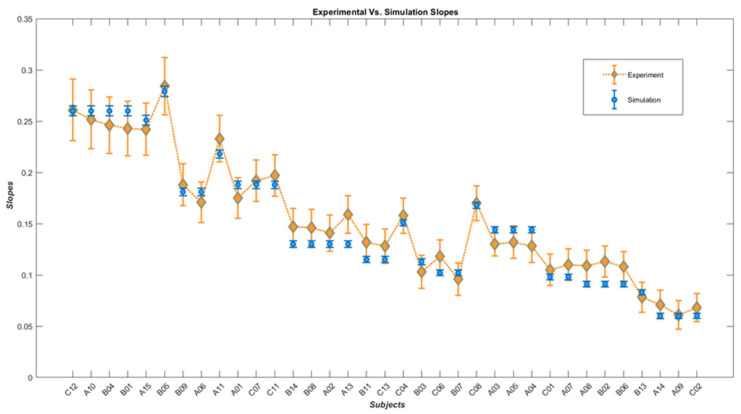
Diamonds represent the slopes of the subjects’ psychometric functions while black dots demonstrate the simulated slopes.

Moreover, a ranked assembly of experimental angular thresholds versus their simulated counterparts is plotted in Figure 6, and the corresponding slopes to the angular thresholds are plotted versus their corresponding simulated slopes in Figure 7. The two figures provide a more discernable comparison between experimental and simulation results.

Correlation analysis shows a significant positive correlation between experimental and simulated angular threshold values, with the Spearman correlation coefficient $r_{s}=0.991,\;p-value=7.08\times 10^{-31}\;(p<0.001)\;$and another significant positive correlation between simulated and experimental slope values with Spearman correlation coefficient $r_{s}=0.963,\;p-value=2.7\times 10^{-20}\;(p<0.001)$. Additionally, to regard the descriptive model’s simulated angular threshold/slope outputs as independent variables to explain experimental human behavior, linear regression modeling was executed, with experimental angular threshold and slope being the dependent variables. More precisely, one linear model that simulated an angular threshold/slope array explains the experimental angular threshold, while the second linear model simulating angular threshold/slope describes the experimental slope. The R-squared and adjusted R-squared values for the angular threshold linear model are 0.965 and 0.963, respectively. Similarly, the linear regression model for the experimental slope model exhibits an R-squared of 0.747 and an adjusted R-Squared of 0.731 as measures for the goodness of fit.

## 5. Discussion

We applied our descriptive risk-averse Bayesian decision-making approach to the third layer and the mutual inhibition method to the fourth layer of the hierarchies, and unlike its predecessor proposed methods (Casile and Giese, 2005; Giese and Poggio, 2003) [2,5], this model showed notable success in simulating human behavior in the sense of mimicking their psychometric function. Therefore, despite all the existing limitations of the model, mirroring the behavior of 11 athletic subjects was accomplished. Moreover, we deem that the model has not yet met its full capacity, and that its potential to encompass the behavior of all the subjects has yet to be implemented. One other future work at hand is to integrate the reaction time into the present model.

It is generally agreed that the human visual system exploits a mixture of all sorts of motion and form cues to detect biological motion and neither the optical flow features nor the form features in and of themselves are adequate for biological motion recognition (Blake and Shiffrar, 2007) [1]. Here, by the proposed model, we aimed to investigate and test the extent of the optic flow features sufficiency to discern a complex biological motion stimulus. The results seem to corroborate the findings in the study of Gliaie and Dotan (2015), which does not find the form cues integral to biological motion detection (Gilaie-Dotan et al., 2015) [13]. Furthermore, our findings are in line with the claim in the Thurman and Lu (2014) study suggesting that the ventral pathway processes the dynamic biological and non-biological forms in the same fashion (Thurman and Lu, 2014) [10].

However, there are limitations to be considered and discussed. One imposed constraint is the fact that each opponent motion neuron in our model only looks at two horizontally abutting receptive fields while in reality some of these neurons are wired to pool the signals from two distant receptive fields, enabling the visual system to process more global relative motions in a moving scene (A. T. Smith and Snowden, 1994) [23]. Furthermore, for the sake of simplification, and as in previous studies, the activity of the local motion neurons were approximated by the computation of the optical flow from the stimulus animation (Casile and Giese, 2005) [5]. Additionally, both the first and second layers have been presumed to be noise free. While the existence of rotation detectors have been substantiated in the human visual system (A. T. Smith and Snowden, 1994) [23], in our model, opponent motion detection level only provides the next level with expansion and contraction cues while rotation cues are an additional source of information from which the decision making parts of the model could benefit. Again, online learning is another capacity that needs to be implemented.

Additionally, it has been shown that executive function deficit leads to slower cognitive processing speed and longer time for completing tasks (Hill, 2004) [39]. Moreover, disorders such as anxiety disorder, major depressive disorder, attention deficit hyperactivity disorder, and autism impair the executive function (Hosenbocus and Chahal, 2012) [40]. Furthermore, one introduced model has suggested that some forms of autism are caused by an increased ratio of excitation/inhibition in sensory, mnemonic and some other systems because of genetics and environmental factors affecting one’s neural system (Rubenstein and Merzenich, 2003) [41]. In our model, the increase in the processing time appearing in large inhibitory gains (which affects the excitation/inhibition ratio of the motion pattern neuronal system) could be construed as noticeable compliance of the present model with current findings.

The present model uses fixed prototypes, parameters, and priors to perceive and make decisions. A more comprehensive model could benefit from online learning and adaptation capacities. To implement such capabilities in the current platform and empower it to detect biological motion through online learning also lies in our plan for future work.

## Figures and Tables

**Table 1:** The parameters’ contribution to the model’s decision-making behavior.

Subjects	Angular Thresholds from Experiment	Angular Thresholds from Simulation	Slopes from Experiment	Slopes from Simulation	Inhibitory Gain (k)	Time Constant (τ)	Noise (δ)
C12	4.041 ± 1.05	**5.209 ± 0.200**	0.261 ± 0.03	**0.260 ± 0.0048**	4	0.0245	0.022
A10	4.176 ± 1.08	**˶**	0.252 ± 0.028	**˶**	**˶**	**˶**	**˶**
B04	4.506 ± 1.1	**˶**	0.246 ± 0.027	**˶**	**˶**	**˶**	**˶**
B01	4.805 ± 1.12	**˶**	0.243 ± 0.026	**˶**	**˶**	**˶**	**˶**
A15	5.321 ± 1.14	**5.448 ± 0.205**	0.242 ± 0.025	**0.251 ± 0.0047**	2	0.033	0.032
B05	5.361 ± 1.04	**5.425 ± 0.193**	0.284 ± 0.028	**0.279 ± 0.005**	4	0.037	0.03
B09	6.602 ± 1.41	**6.871 ± 0.268**	0.188 ± 0.02	**0.181 ± 0.0036**	4	0.025	0.034
A11	6.637 ± 1.52	**˶**	0.171 ± 0.019	**˶**	**˶**	**˶**	**˶**
A06	6.609 ± 1.21	**6.556 ± 0.232**	0.233 ± 0.022	**0.218 ± 0.004**	8	0.033	0.032
A01	7.000 ± 1.51	**7.228 ± 0.263**	0.175 ± 0.019	**0.188 ± 0.0036**	8	0.03	0.034
*C07*	7.097 ± 1.42	**˶**	0.192 ± 0.02	**˶**	**˶**	**˶**	**˶**
C11	7.165 ± 1.39	**˶**	0.197 ± 0.02	**˶**	**˶**	**˶**	**˶**
B14	7.692 ± 1.79	**7.664 ± 0.363**	0.147 ± 0.017	**0.130 ± 0.0031**	1	0.024	0.026
B08	7.753 ± 1.8	**˶**	0.146 ± 0.017	**˶**	**˶**	**˶**	**˶**
A02	7.837 ± 1.86	**˶**	0.141 ± 0.017	**˶**	**˶**	**˶**	**˶**
A13	7.873 ± 1.69	**˶**	0.159 ± 0.018	**˶**	**˶**	**˶**	**˶**
B11	8.132 ± 2	**8.509 ± 0.421**	0.132 ± 0.017	**0.115 ± 0.003**	1	0.024	0.028
C13	8.594 ± 2.08	**˶**	0.128 ± 0.016	**˶**	**˶**	**˶**	**˶**
C04	9.173 ± 1.77	**9.275 ± 0.337**	0.158 ± 0.017	**0.151 ± 0.003**	16	0.025	0.034
B03	9.191 ± 2.64	**9.198 ± 0.438**	0.103 ± 0.016	**0.113 ± 0.0029**	2	0.024	0.028
C06	9.543 ± 2.34	**9.818 ± 0.496**	0.118 ± 0.016	**0.102 ± 0.0028**	2	0.024	0.03
B07	9.589 ± 2.86	**˶**	0.096 ± 0.015	**˶**	**˶**	**˶**	**˶**
C08	9.747 ± 1.69	**9.791 ± 0.313**	0.170 ± 0.017	**0.168 ± 0.0031**	32	0.033	0.032
A03	10.49 ± 1.56	**11.131 ± 0.376**	0.130 ± 0.011	**0.144 ± 0.0028**	32	0.025	0.34
A04	10.801 ± 2.2	**˶**	0.132 ± 0.015	**˶**	**˶**	**˶**	**˶**
A07	10.843 ± 2.25	**˶**	0.128 ± 0.015	**˶**	**˶**	**˶**	**˶**
A05	10.77 ± 2.71	**10.696 ± 0.529**	0.105 ± 0.015	**0.098 ± 0.0028**	4	0.024	0.028
C01	10.83 ± 2.6	**˶**	0.110 ± 0.015	**˶**	**˶**	**˶**	**˶**
A08	12.132 ± 2.75	**12.315 ± 0.606**	0.109 ± 0.015	**0.091 ± 0.0027**	8	0.024	0.028
B02	12.173 ± 2.67	**˶**	0.113 ± 0.015	**˶**	**˶**	**˶**	**˶**
B06	12.525 ± 2.81	**˶**	0.108 ± 0.014	**˶**	**˶**	**˶**	**˶**
B13	12.86 ± 3.93	**12.258 ± 0.664**	0.078 ± 0.014	**0.083 ± 0.0027**	2	0.024	0.034
A14	16.617 ± 4.88	**17.978 ± 1.105**	0.071 ± 0.013	**0.06 ± 0.0025**	8	0.024	0.036
A09	17.194 ± 5.84	**˶**	0.061 ± 0.013	**˶**	**˶**	**˶**	**˶**
C02	17.787 ± 5.35	**˶**	0.068 ± 0.013	**˶**	**˶**	**˶**	**˶**

Table 1: by tuning the k, τ and δ Parameters the angular thresholds (75%) and the slopes of athletes’ psychometric functions have been simulated (symbol ˶ indicates that the quantity in that placeholder is the same as the quantity above it).

## Data Availability

Not applicable.
